# A Scoping Review on Long COVID-19: Physiological and Psychological Symptoms Post-Acute, Long-Post and Persistent Post COVID-19

**DOI:** 10.3390/healthcare10122418

**Published:** 2022-11-30

**Authors:** Krishna Mohan Surapaneni, Manmohan Singhal, Sofia Rani Saggu, Ashruti Bhatt, Priya Shunmathy, Ashish Joshi

**Affiliations:** 1 School of Pharmaceutical & Population Health Informatics, Faculty of Pharmacy, DIT University, Mussoorie, Diversion Road, Makka Wala, Dehradun 248 009, Uttarakhand, India; 2 SMAART Population Health Informatics Intervention Center, Foundation of Healthcare Technologies Society-Panimalar Medical College Hospital & Research Institute, Varadharajapuram, Chennai 600 123, Tamil Nadu, India; 3 Department of Biochemistry, Medical Education, Molecular Virology, Research, Clinical Skills & Simulation, Panimalar Medical College Hospital & Research Institute, Varadharajapuram, Chennai 600 123, Tamil Nadu, India; 4 Foundation of Healthcare Technologies Society, 321, 322 & 323 Third floor, Somdatt Chamber-29, BhikajiCama Place, New Delhi 110 066, India; 5 School of Public Health, The University of Memphis, Memphis, TN 38152, USA

**Keywords:** post COVID-19, persistent symptoms, fatigue, long COVID-19, post-acute sequalae

## Abstract

Background: The identification of persistent symptoms of chronic/long COVID-19 is crucial in understanding the management of long haulers of post COVID-19. Methods: Pub Med (Medline) database was scoped for original articles based on a search strategy related to the objectives. The selected articles post-screening were analyzed for variables relating to chronic/long COVID-19. Results were analyzed using descriptive statistics. Results: A total of 33 studies were reviewed. A total of 60% of the studies were observational studies and most of them were from high income countries. Almost half of the studies were in phase 3 of post-COVID-19, i.e., symptoms lasting >24 weeks. Among the physiological and psychological symptoms studied, fatigue, dyspnea, cough, headache, memory loss, depression, brain fog and lack of concentration were found to be the most frequently reported symptoms. Excessive sleep, constipation and neuropathic pain were among the least reported symptoms. Prior hospitalization, the female gender was found to be a risk factor. Limitations were reported by all studies. Conclusions: The major physiological and psychological symptoms of long COVID-19 have been explained with risk factors and its impact on patients’ lifestyles. The findings of this review hope to facilitate clinicians to draw conclusions to manage the long-term effects of post/chronic COVID-19.

## 1. Introduction

The Corona virus disease or the COVID-19 outbreak that started in Wuhan in China in December 2019 shook the global healthcare system and posed a threat to the healthy survival of people worldwide. It was declared a pandemic in March 2020 and the first case of the COVID-19 virus in India was reported in 2020 January [1,2]. Over the years, many other devastating infections, such as Severe Acute Respiratory Syndrome Corona Virus (SARS-CoV) and Middle Eastern respiratory syndrome corona virus (MERS-CoV), have emerged, with SARS-CoV and MERS-CoV infections being more fatal than SARS-CoV-2. However, the COVID-19 pandemic was different and created panic among people as it showed high viral shedding and a high proportion transmission rate compared to MERS or SARS [3]. Worldwide, the Corona virus disease (COVID-19) virus has created significant risks to people’s mental and physical health. Although patients and health staff receive psychological care, public mental health in general demands major attention. Furthermore, and also more generally, the prevalence of mental disorders, especially depression, increased significantly with the onset of the pandemic. This should not come as a surprise, since the pandemic is accompanied by numerous psychological stress factors: the fear of the disease, of suffering and the death of relatives and friends, the fear of job loss, social tensions due to lockdown, working from home and remote schooling [4].

Physically, patients suffer from fever, tiredness, accompanied by dry coughs without mucus or phlegm expectoration and diarrhea with muscle discomfort, disorientation, headache, sore throat, rhinorrhea, chest pain, sputum production and nausea and vomiting being among the less prevalent symptoms [5]. These signs and symptoms are distinctively different from other diseases in terms of origin, onset, duration, progression, severity and response to traditional treatment, and thus cannot be explained in terms of other diseases. Most people who develop COVID-19 fully recover, but current evidence suggests that approximately 10–20% of people experience a variety of mid- and long-term effects after they recover from their initial illness. These mid- and long-term effects are collectively known as the post COVID-19 condition or “long COVID-19”. “Long COVID-19” refers to the existence of symptoms weeks or months after contracting the SARS-CoV-2 infection, regardless of the viral state [6]. The long COVID-19 or post COVID-19 condition is defined as the illness that arises in patients who have a history of suspected or confirmed SARS-CoV-2 infection, usually within three months of the commencement of COVID-19, with symptoms and consequences lasting at least two months [7]. Different nomenclatures as well as the timeline of defining post COVID-19 or long COVID-19 are available. A common consensus is the persistence of symptoms beyond 2 months (>8 weeks) [8,9,10]. With regards to identifying persistent symptoms, an alternative diagnosis cannot explain the signs and effects of the post COVID-19 syndrome (as per the WHO). Although such alteration is mainly reported in severe and critical disease survivors, the lasting effects also occur in individuals with a mild infection who did not require hospitalization [11].

The major symptoms of post COVID-19 illness that influence peoples’ quality of work and everyday lifestyle are performance and perceived fatigue, shortness of breath, problems related to memory, sleep and concentration, constant cough, chest ache, difficulty in speaking, muscular pain, anosmia, ageusia, psychological manifestations and febrile illness. A possible way to safeguard oneself from post-COVID-19 is to be preventive towards acquiring the COVID-19 infection by adhering to all standard operating protocols. The effect of vaccination on reducing the post COVID-19 symptoms is under research. Even though transmission of post COVID-19 symptoms is improbable, one should seek immediate medical advice for managing and treating the same. Presently, specific medications or treatment strategies have not been proposed for the post COVID-19 condition. However, effective rehabilitation and holistic medical assistance are considered to be the typical symptoms management protocol for patients experiencing post COVID-19 syndrome [12]. A broad overview of all the possible longstanding effects of COVID-19 is still needed. Therefore, our study aimed to perform a scoping review of peer-reviewed studies to identify the prevalence of all the symptoms in post COVID-19 reported up to the month of September 2022.

The objective of this scoping review is to conduct a scoping search on the long-term consequences of COVID-19 on the physiological and the psychological health of COVID-19 long haulers. A record of symptoms should reveal which aspects of the sequalae are common, which symptoms are secondary to a complicated form of COVID-19. A study on the long-term effects of COVID-19 even in physiological systems such as respiratory, neurological, etc. is required to understand treatment options that might also be useful in the future when managing any new disease or emergency conditions.

### Objectives

This scoping review seeks to

- critically analyze and comprehend the available literature on post/long COVID-19 symptoms.
- examine the long-term consequences of COVID-19 on the psychological and physiological health of long haulers of COVID-19.
- report on the common physiological as well as psychological symptoms and their prevalence among patients recovered from COVID-19.
- identify the factors that are associated with the severity of long COVID-19 symptoms.
- identify the population at risk and complications that cause prolongation of COVID-19 symptoms.
- discuss the potential impact of long COVID-19 symptoms on health andquality of life.

## 2. Methodology

The systematic methods used for retrieving information from scientific the literature and reporting on the available data are explained in Table 1.

**Table 1:** Methodology.

S.No	Major Step	Objective	Method	
2.1	Search strategy	The review will seek to identify existing research literature that may be used to support evidence-based practice.	The present scoping review used the iterative five-stage methodological framework of Arkskey and O’Malley that included the following steps: (i) defining the research topic, (ii) identifying relevant research papers, (iii) selecting the study, (iv) charting the data, and (v) collating, reporting and summarizing the findings [13].	
		Database Used	Pub Med	
		Key words and Medical Subject Headings (MeSH):	Long COVID, Long haul COVID, Post-acute COVID-19, post-acute sequalae of SARS-CoV-2, Chronic COVID-19, Chronic COVID-19 syndrome, Corona virus, long term effect, long-term sequalae, signs and symptoms and mental health.	
2.2	Identification of relevant studies	To conduct a thorough literature search on the physiological and psychological symptoms of post/long COVID-19	Original articles published from 2020 to 2022 as researched in the MEDLINE database of Pub Med. The search and extraction of data were conducted in the month of September 2022. The inclusion and exclusion criteria are as follows:	
2.3	Inclusion Criteria	The articles that were included for this review were selected based on the inclusion criteria mentioned in Methods.	Criteria	Inclusion Eligibility
			Theme of study	Studies related to the long-term effects of Covid-19, or post-sequalae of COVID-19 or post COVID-19.
			Study Design	Original studies including Classical Article, Clinical Study, Clinical Trial, Multicenter Study and Observational Study.
			Grey Literature	Preprints, case reports (Grey literature) specific to the objective
			Publication Journal	The articles should have been published in a pre-reviewed journal.
			Language	Articles in the English language
2.4	Exclusion Criteria	The articles that were excluded from selection for this review was based on the exclusion criteria as previously mentioned.	Criteria	Excluded articles
			Theme of study	Those irrelevant to COVID-19
			Study Description	Studies not specific to objectives, studies reporting on patient characteristics and studies among participants that did not have a prior COVID-19 infection
			Full text access	Unavailable full text
2.5	Selection of sources of evidence	To select the appropriate articles from the results of the search	Using the Preferred Reporting Items for Systematic reviews and Meta-Analysis (PRISMA) extension for Scoping Reviews/(PRISMA-ScR) Checklist, we retrieved 1075 articles from our search (Figure 1). Title screening—958 articles were excluded as they were not in the context of post-COVID-19 but were included for Abstract screening—117 articles Full-text screening—42 included and 75 articles were excluded. (The reasons for exclusion are mentioned in Figure 1 in the results section). Included articles—33 The studies for this review were chosen in two stages. 1. The titles and abstracts of all retrieved studies were evaluated by one reviewer and then checked by another reviewer in the first stage. 2. The same reviewers read the entire texts of papers included in the first step independently in the second step. Any differences between the two reviewers were resolved by consulting a third reviewer.	
2.6	Data charting	To comprehensively review the articles included for the study and extract the variables under different categories for critical analysis	Data variables identified from the full text of articles are: Year of study, Study objectives, Keywords mentioned, Study design, study location, Study Population, Study sample size, Inclusion and exclusion criteria, COVID-19 Phase studied, Follow up, Symptoms reported, Major findings and Study limitations.	

## 3. Results

The results obtained from the articles included were analyzed and reported using the following strategy described in Table 2 and the extracted data is represented in Table 3 and Table 4.

### 3.1. Study Characteristics

The articles were all assessed by the following categories: location, population group and time point after COVID-19, study tools, predominant systems studied and their corresponding symptoms. The study characteristics have been summarized in Table 4.

### 3.2. Types of Articles Included

Out of the 33 articles included in the study, all articles were original articles. Nineteen were observational studies, ten were multi-centric studies, two were clinical trials and one each from a cross-sectional and cohort study were studied. Editorials and review articles were not included in the study (Table 5).

### 3.3. Timeline Brief

The publications of long COVID-19/chronic COVID-19 were selected through a thorough search of database. In total, 14 articles from the year 2021 followed by 19 articles in 2022 were included in this review. 

### 3.4. Study Location

**Figure 2:**
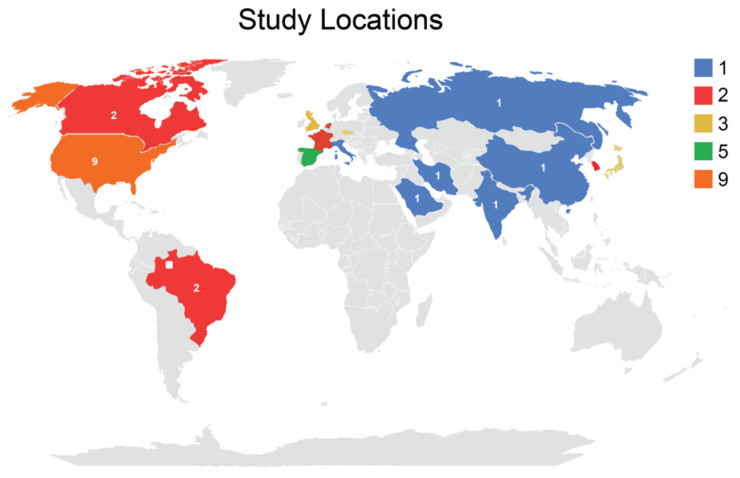
Study locations.

Among the 33 studies, the majority were conducted in the USA (9 studies), followed by Spain (5 studies), UK (4 studies), Denmark (2 studies) and Italy (2 studies).Other countries include India, England, France, Brazil, Canada, Russia, Iran, Belgium, Switzerland, Saudi Arabia and China (Figure 2).

### 3.5. Income Classification

The countries when classified according to their economic levels show that 81.8% (*n* = 27) of the studies were from high-income economies, 12.12% (*n* = 4) from upper-middle income economies and 6% (*n* = 2) of the studies were carried out in low-income countries (Table 6).

### 3.6. Study Population

Among the 33 articles, 30 (91%) were related to the adult population (>18 years), two (6%) included children (one study included only children, and the other included children and adolescents), and one (3%) study was based on the elderly population (Table 5). The study, which included the elderly population, consisted of 2242 participants aged from 64 to 73 years (mean being 68 years) (ID18) [31]. Among the studies conducted among children, one study consisted of children aged from 8–18 years and had 53 participants (ID 10) [23] and the other study included children and adolescents 0 to 18 years (ID17) [30]. In the other studies involving the adult population, the most common range was from 56–64 years (13 studies, 43%), followed by 41–49 years (6 studies, 20%). The study by Asadi-Pooya et al. included highest number of participants *n* = 4681 (ID 6) [19]. The study with the least number of participants was the study by Krishnan et al., with 20 participants (ID27) [40].

### 3.7. Number of Participants

Among all the studies, there was only one (3%) study which consisted of 4681 participants. The participants were all adults, and the mean age was 52 years. (ID6) [19]. Two (6%) more studies had 2649 and 1969 participants (ID 5 and 14, respectively) [27,28], who were also adults. The other studies involving the adult population ranged from 24 to 1296. Table 7 denotes the distribution of the study population.

### 3.8. Keywords

**Figure 3:**
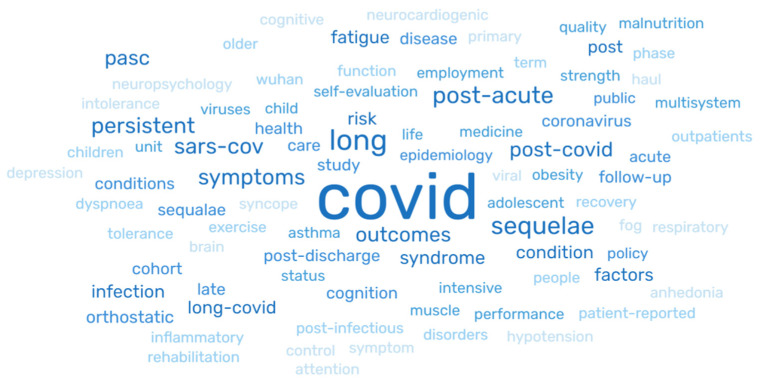
Keywords among the study results.

Of the 33 studies reviewed, eight (24%) studies did not mention keywords. The major keywords identified in the studies are presented in Figure 3. The most recurring word is highlighted in darker hues of blue (COVID-19, long, post-COVID-19, post-acute, sequalae) and the least recurring words (brain, haul, phase, Wuhan) are in lighter hues of blue (Figure 3).

### 3.9. Phase of COVID Study

**Figure 4:**
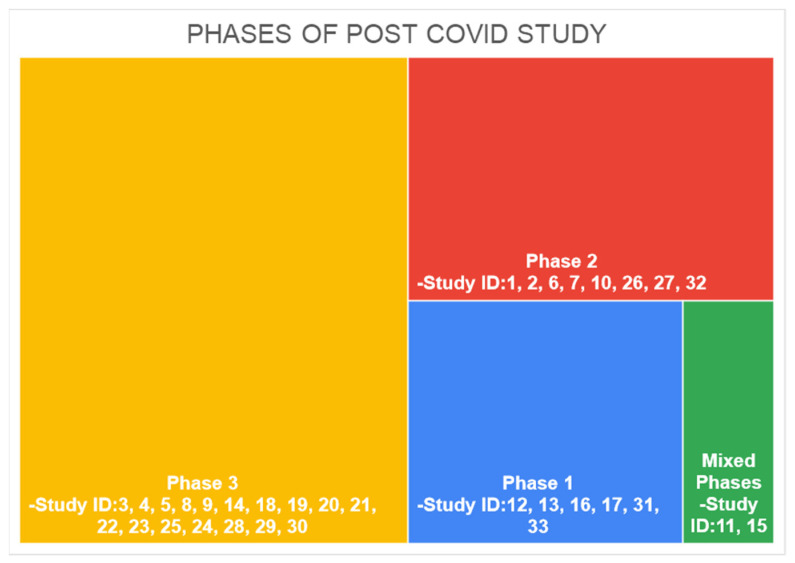
Phase of Post-COVID-19 Study.

Long COVID-19 studies are differentiated by their study of persistent symptoms. Phase 1, called “acute post-COVID-19” symptoms, consisted of symptoms presented between week 5 and week 12 since COVID-19 diagnosis; Phase 2 was called long post COVID-19 and presented symptoms from week 12 and week 24 since COVID-19 diagnosis; and Phase 3, or “persistent post COVID-19 symptoms”, consisted of symptoms lasting more than 24 weeks. The time period for assessing symptoms in participants of studies included in our review is presented in Figure 4.

The number of studies conducted in phase 1 of the COVID-19 symptom phase was *n* = 6 (18%), Phase 2 was eight studies (24%) and Phase 3 was 17 studies (51%). Two studies (6%) reported symptoms from the two phases. A PHOSP-COVID Collaborative Group conducted the study in Phases 2 and 3 (ID11) [24], whereasthe study by Silverberg et al. reported symptoms from all phases (ID15) [28].

### 3.10. System Studied

Almost all the studies identified symptoms regarding the musculoskeletal system, followed by the respiratory system, cardiology, neurosensory disorders and the gastro-intestinal system. Within the musculoskeletal system, the common, persistent symptoms were chronic fatigue, myalgia, arthralgia, intolerance to exertion and thoracic and rib pain. Among those studies, eight studies gave importance to exertional intolerance (ID 7, 12, 20, 23, 27, 28, 29) [20,25,33,36,40,41,42]. In cardiology, chest pain and dyspnea were the major focus areas. Imaging studies and other clinical investigations were used by few studies to assess the already existing co-morbidities and to find persisting symptoms related to COVID-19. Those co-morbidities were either used as an exclusion factor or to identify those conditions as a risk factor for developing COVID-19 persistent symptoms. The integumentary system (skin, hair, glands, nerves and blood vessels) was also studied by a few studies. Among them, hair loss was identified in eight studies (24%), rashes/other skin disorders in five studies (15%) and anemia in three studies (9%). Certain studies also gave importance to sensory functions such as numbness and vision impairment. Some studies focused specifically on certain characteristics such as functional and physical impairment, multi-organ impairment, neurological manifestations, anthropometry, biological markers, immune status, health-related quality of life and general mental state. There were several articles which were based on clinical laboratory tests and imaging features. Out of these, pulmonary function tests were used in three studies (ID5,8,12) [18,21,25], antibody assessment in two studies (ID 16,21) [29,34] and immunoassays were used as a tool in one study (ID 10) [23]. Other imaging studies such as CT, MRI and ECG were seen in a few studies as well. Two studies (6%) were focused mainly on neurocognitive profiles. Almost all the studies used valid study tools and criteria for the assessing persistent symptoms. For assessing one of the most common and persistent symptoms, dyspnea, tools such as Dyspnoea-12 (ID 9, 19) [22,32], Self-Evaluation of Breathing Questionnaire (SEBQ) (ID 12) [25], The King’s Brief Interstitial Lung Disease questionnaire (ID 33) [46], etc. were used. For assessing disability, World Health Organization Disability Assessment Schedule was used by a few studies.

For measuring the health quality of life, a few studies had used EuroQol: 5 dimension, 5 level (EQ-5D-5L) scale (ID 1,3,11,19, 22,25) [14,16,24,35,38]. One study had also used the Post-Traumatic Stress Disorder Checklist for the Diagnostic and Statistical Manual of Mental Disorders (PCL-5) for specifically assessing stress-related long COVID-19 symptoms (ID 32) [24]. Most of the studies had also used validated self-assessment questionnaires and other data gathering forms.

### 3.11. Symptoms Presented

Of all the forty-five persistent symptoms evidenced from the studies, a total of 36 symptoms were physiological symptoms and 8 were psychological symptoms.

### 3.12. Physiological Symptoms Presented

All studies from our review reported physiological symptoms (*n* = 33, 100%). The main long COVID-19 persistent symptoms identified were as follows: performance and perceived fatigue (*n* = 29, 87%), shortness of breath (*n* = 26, 78%), cough (*n* = 15, 45%), headache (*n* = 13, 39%) and insomnia (*n* = 13, 39%), followed by other symptoms such as myalgia, chest pain, palpitations, etc., which are presented in Table 8.

### 3.13. Psychological Symptoms

From our review, psychological symptoms were reported in 33%, *n* = 11 studies. Psychological symptoms identified in studies are brain fog, depression and mood disorders, memory loss/forgetfulness, confusion, anxiety, post-traumatic stress, concentration/cognitive impairment, and slowed down thinking. Among the psychological symptoms mentioned in Table 6, the most common persisting symptom identified was memory loss (9 studies, 27%), followed by depression (7 studies, 21%). The distribution of psychological symptoms identified has been presented in Table 9.

### 3.14. Major Findings

Our comprehensive analysis puts forth the findings that recently there has been a rapid increase in population with long COVID-19. The occurrence of long COVID-19 may be influenced by other factors than those producing acute illnesses. Post COVID-19 symptoms were more profound among females, hospitalized patients and unvaccinated individuals. Furthermore, symptoms were identified to persist for a prolonged duration if they start appearing within the 5th to 12th week after the diagnosis of COVID-19 infection. Data also showed that acute COVID-19 symptoms may not predict mental sequalae accurately in this phase, prompting further research into the links between acute sickness experience and PASC. An immunoglobulin (Ig) pathological mechanism distinct from merely increased inflammation and immune activation can identify patients at risk for developing PACS.

During phase 2, the majority of studies reported links between delayed recovery from COVID-19 to the presence of co-morbidities such as hypothyroidism or respiratory problems, which influence the persistence of long COVID-19, even their cognitive defects. The longitudinal impact on quality of life parameters in children highlights the importance of close monitoring of children and adolescents by the clinical team after COVID-19. An important finding in phase 3 post COVID-19 is the presence of symptoms more at one year post COVID-19, more so than that in phase 1. Most patients reported symptoms at 6–8 months commencing from the time of discharge. Phase 3 studies also reported a higher risk of post COVID-19 in females. Post COVID-19 symptoms, especially after one year of infection, substantially affect the daily life of the person. Delayed onset of symptoms such as cough and arthromyalgia were reported to be more common at 12 months than at 4 months after discharge. Post COVID-19 syndrome was assessed to be a combination of perceived and performance fatigability, physically slowing down, poor sleep, breathlessness, joint pain, slowed down thinking, short-term memory loss, limb weakness, change in smell or taste, muscle weakness, cough, headache and stomach pain. Unfortunately, the recovery and treatment outcomes were found to be sub-optimal among women. The burden of symptoms along with reduced exercise capacity and large decrements in health related quality of life were reported in those patients, 1 year after hospital discharge.

Post COVID-19 syndrome caused mild organ impairment in the heart, lungs, kidneys, liver, pancreas and spleen. The severity of impairment was associated with older age, non-white ethnicity, large liver volume, pancreatitis, accumulation of fat in the liver and pancreas and cardiac dysfunction. Respiratory symptoms such as difficulty in breathing and exertion that causes perceived fatigability and performance fatigue due to inadequate oxygen supply were found to be more dominant in the post COVID-19 phase among patients who had co morbidities such as hypothyroidism and hypoxia at the time of acute COVID19 infection. Many studies have put forth the fact that psychological symptoms such as memory loss, depression, mood swings, brain fog, anxiety, difficulty in concentration, impairment in cognition, confusion, decreased thinking ability and post-traumatic stress affect the quality of life in patients who suffered from COVID-19 infections. These symptoms persist for a longer period of time among hospitalised patients. Effective rehabilitation and management is necessary to revert the psychological symptoms and bring the patient back to a normal lifestyle. A major post COVID-19 symptom is loss of appetite, which leads to malnutrition and anaemia among the patients. Intake was food was also affected by anosmia and ageusia caused by COVID-19 infection.

Most of the post COVID-19 symptoms depend on the individual parameters and lifestyle practices. Although a majority of the patients experienced insomnia as a post COVID-19 symptom, few experiments have proven that excessive sleepiness could also to be a symptom of post COVID-19. Many patients with acute COVID- 19 illness were found to be suffering from diarrhea in the post COVID-19 stage. Contrary to this, studies have also revealed that constipation is a sign of post COVID-19. Hence, there is no clarity regarding standardisation of post COVID-19 symptoms. It is also important to understand that there are several limitations in the studies included for this review. As it is vital to take into account all these limitations to analyse better outcomes and identify the gaps in research, comprehensive steps can be taken with future studies to fill the voids in the literature. A summary of major limitation of the studies included for this review are presented in Table 10 below.

## 4. Discussion

The current scoping review aimed to examine the long-term effects of COVID-19 on psychological and physiological health in people who had been diagnosed with COVID-19 and to investigate the evidence of post COVID-19 study characteristics using the findings of various studies conducted globally on the chronic effects of post COVID-19. The key characteristics are discussed below.

### 4.1. COVID Phase

In the present study, chronic post COVID-19 studies were differentiated by their period of study and persistence of symptoms. As it is currently recognized that post COVID-19 symptomatology is more variable and more complex than predicted, which may explain why there is no unanimity in the definition of post COVID-19 [47], there exists many terminologies, a few of which are presented comprehensively by Yong et al., [48]. We have used the timeline model of Fernández-De-las-peñas, C et al., where they defined the following: Phase 1, called “acute post COVID-19” symptoms, consisted of symptoms presented in between weeks 5 to 12 weeks since COVID-19 diagnosis; Phase 2, called long post COVID-19, presented symptoms from weeks 12 to 24 weeks since COVID-19 diagnosis; and Phase 3, or “persistent post COVID-19 symptoms”, consisted of symptoms lasting more than 24 weeks [10].

Among all the studies, there were six studies conducted during Phase 1, eight studies during Phase 2 and seventeen studies during Phase 3. There were two studies that included mixed phases. One was conducted during Phase 2 and 3 [24] and the others were conducted during all the three phases [28]. Information on period of inclusion and follow-up helped to clearly distinguish the persistence of symptoms of COVID-19. There were a few studies that consisted of the follow-up period for variable lengths of time. Among them, a study conducted by Darcis Gilles et al., assessed the patients post-discharge, 3 months and 6 months after COVID-19 infection [20]. Similarly, another study conducted by The PHOSP-COVID Collaborative Group (UK) assessed the patients at 5-month and one-year intervals [24]. Fernández-de-Las-Peñas, C et al., evaluated symptoms for outpatient participants at the time of acute infection, at 30 days, 60 days and 90 days [37].

### 4.2. Study Population

Among the 33 articles, 30 (91%) were related to the adult population (>18 years), 2 (6%) included children (one child, one child and adolescents) and 1 (3%) study was based on the elderly population. Almost all the studies included patients who were with either Reverse Transcriptase Polymerase Chain Reaction (RT- PCR) confirmed SARS-CoV-2 infection or clinically confirmed infection by physicians or laboratory tests.

In a study by Finkk et al., which was conducted on children, asymptomatic participants with the disease and those who had severe cognitive dysfunction were not included, which might have affected the study findings with regard to long-term COVID-19 symptoms that may arise in asymptomatic individuals, especially in children [23]. According to a study by Lamontagne et al., only right-handed participants (aged between18 and 60 years) were included, and no explanation was given for the inclusion criteria. Though the proportion of right-handed individuals are high in the community, this study still fails to represent the left-handed population [46].

### 4.3. Signs and Symptoms

Performance and perceived fatigability/tiredness/weakness (*n* = 29, 87%), shortness of breath/dyspnea/breathlessness (*n* = 26, 78%), cough (*n* = 15, 45%), headache (*n* = 13, 39%) and insomnia (*n* = 13, 39%) were the most reported physiological symptoms. The least reported symptoms were sexual dysfunction, excessive sleepiness, neuropathic pain, constipation, loss of functional status, rib pain/thoracic pain, dysuria, haematuria, oliguria and glycaemia/renal problems, all of which were reported by one study each (*n* = 1, 3%). Among psychological symptoms reviewed, memory loss/forgetfulness was reported in nine studies (27%), followed by brain fog (*n* = 6, 18%), anxiety (*n* = 6, 18%), concentration/cognitive impairment (*n* = 6, 18%), confusion (*n* = 3, 9%), slowed down thinking (*n* = 2, 6%) and post-traumatic stress (*n* = 1, 3%). Our findings in this review are consistent with findings of other investigators, who reported symptoms affecting physical and mental health. They reported post COVID-19 psychological and neuropsychological concerns (anxiety and depression, PTSD, sleep and cognition deficits), even in people who had never had a mental health condition, which is similar to our findings [49, 50]. In another study conducted by Van Kessel et al., it was found that psychological, cognitive and social symptoms were associated more with post-acute COVID-19 syndrome and less with long COVID-19 haulers. The study also mentioned that social symptoms were mainly studied in qualitative studies [51].

In a study conducted by Dennis Andrea et al., which was focused on multi-organ impairment in COVID-19 patients, there were two major findings. The first one was that in low-risk individuals, there were chronic symptoms and mild impairment found in the heart, lung, liver, kidney and pancreas compared with healthy controls. Additionally, the second finding was that cardiac impairment was more common in severe persistent COVID-19 syndrome [14]. According to Bellan et al., who assessed the persistence of symptoms from severe COVID-19 one year after hospital discharge, some symptoms, such as cough and arthromyalgia, were even more common at 12 than at 4 months after discharge [17]. According to Gérard et al., in a study which was conducted to assess the neglected components of long COVID-19 syndrome such as malnutrition, showed that at day 30 after COVID-19 infection, 138/288 of the patients (48%) presented with persistent malnutrition (33%) and subjective functional loss (26.3%). Furthermore, it was noted that at 6 months, 15% of the initial participants remained malnourished despite nutritional counseling and dietary guidance, oral nutritional supplements or relocation to rehabilitation centers [21]. In a study conducted by Twomey et al., the main factors associated with chronic fatigue and Post Exertional Malaise (PEM) in people living with long COVID-19 was studied. The study found that for more than 60% of those experiencing PEM in their study sample, it had been more than 40 weeks since their confirmed/suspected SARS-CoV-2 infection [25]. According to Kikkenberg et al., children who had a history of SARS-CoV-2 infection in all age groups from 0 to 14 years reported a higher prevalence of long-lasting symptoms compared with age-sex-matched controls, and, among the oldest respondents, more females than males had long-lasting symptoms [30]. In our review, the most frequent systems affected were the respiratory, systemic, neurological, mental health and dermatological health systems, with research by Moktari et al., and Subramanian et al., confirming these findings [52,53].

### 4.4. Risk Factors, Major Findings and Limitations

A study from our review by Naik Shivdas et al., stated the associated risk factors for long COVID symptoms were severity of the COVID-19 infection (severe/moderate) and hypothyroidism [15]. Similar findings in cross sectional studies by Burekovic et al., and Lui et al., who reported hypothyroidism as a risk factor and as a consequence of COVID-19 infection [54,55]. In patients with co morbidities such as hypothyroidism, persistent musculoskeletal and respiratory complaints were found were commonly found [15,53]. According to Dennis Andrea et al., even non-hospitalized individuals, or 10% of those infected, have had persistent symptoms related to COVID-19 [14].

According to Faten et al., persistent COVID-19 symptoms such as loss of smell/taste, shortness of breath and fatigue are persistent symptoms which mainly affected social and work-related activities [29]. According to Han et al., persistent symptoms were significantly associated with patients with poorer long-term health status, poorer quality of life and those who were experiencing severe psychological distress [35]. Persistent symptoms were found to be substantially related to poorer long-term health, lower quality of life and psychological distress. An important finding from our review is that female sex is one of the major risk factors for persistent long COVID-19 symptoms [27,30,36,37,45]. A few cross-sectional studies that report similar findings, such as a study by Peghin et al., identified female gender (OR 1.55) as a potential independent risk factor for long COVID-19; a review by Subramanian et al. and a review by Shanbehzadeh also reported the association of being female with an increased risk of reporting symptoms 12 weeks post acquiring COVID-19 [50,53,56]. Notably, fatigue was encountered more by the females compared to males. This refers to both perceived and performance fatigability. Factors such as stress, anxiety, depression and physical pain contribute to fatigue in women. Domestic stress such as marital and family stress caused more depression and mental strain among homemakers, whereas social discrimination, need for financial stability during pandemic, safety concerns and family support caused stress and depression among working women and single women. Furthermore, increased workload, at home or in the workplace, caused physical tiredness and a greater perception of pain among women than men, which resulted in increased fatigability among women compared to men [57]. The majority of the studies mentioned limitations such as recall bias, selection bias, etc. in their study. However, this was not a major limitation in the ten (30%) multicenter studies that included various hospitals and participants from different parts of the countries and globally.

### 4.5. Impact of Long COVID-19 on Health and Quality of Lifestyle

It has been estimated that 2 months after the onset of COVID-19 disease, 87.1% of the patients discharged still experience at least one of the symptoms of long COVID-19, and nearly 55% experience multiple symptoms, such as difficulty in breathing, chest pain and tiredness, all of which affect their quality of life [58]. Here, it is also important to identify the cause of the symptom and confirm the underlying pathogenesis. Physicians and medical practitioners could suggest doing some biochemical and radiological tests to confirm previous infection in response to persistent symptoms. These include blood investigations monitoring blood pressure, heart rate and oxygen levels. Sometimes, a chest X-ray would be needed for a precise diagnosis. There is no standard time identified for the recovery from long COVID-19; most people return to their normal activities in less than 12 weeks, but for some people the symptoms can persist for longer periods of time, hindering their daily activities and quality of work [59]. Treatment options for long COVID-19 depend on the severity and duration of people experiencing the symptoms. Whereas people who have milder manifestations need not require any treatment, people with more severe and persistent signs should be treated by a holistic approach with health specialists for management and effective treatment options. It has been found that for most people, the health outcomes are optimal and a return to normal activities without any trace of other symptoms was reported 9 months after COVID-19infection. However, individuals who are proficient in sports and other forms of physical exercise, individuals who are highly functioning and young individuals who are considered high performing were previously found to improve at a slower rate, which affects their ability to work exercise and socialise [60]. With these people, monitoring the signs and managing the symptoms is highly important to prevent any distressing outcomes and promote better healing [60].

### 4.6. Knowledge Gaps

Although much of the research carried out explores long COVID-19 and its potential causes, there exists a paucity in knowledge regarding the management and cure of these persistent symptoms. The persisting signs and symptoms of post/long COVID-19 is characteristically different from other diseases with respect to onset, duration, progression and underlying health conditions. Of the many mechanisms, oxidative stress is identified to be one of the causes for the long haul that significantly reduces the antioxidant level in the body. In addition, an elevated calcium to magnesium ratio has been reported in these patients. Since the Ca:Mg ratio is crucial for glutamate and GABA balance, many of the gastrointestinal and urological symptoms can be manifested. However, more detailed explanations and in-depth analysis should be made to confirm the above statement so that appropriate diagnostic methods and effective treatment regimens can be suggested for patients with long COVID-19 illness [61]. Furthermore, we know that vaccination emerged as an industry breakthrough in lowering the risk of acquiring COVID-19 infection and breaking the transmission cycle. However, the effectiveness of COVID-19 vaccinations on long COVID-19 and whether it reduces the risk of acquiring long COVID-19 and reduces the duration and severity of the persisting symptoms is still unclear [62]. An international survey analysing the impact of COVID-19 vaccinations on long COVID-19 symptoms suggested that vaccines plays a beneficial role in improving the symptoms of patients who are suffering from post-COVID-19 illness [63]. However, it has not been substantiated with evidence and, hence, stresses the importance of clinical trials and future studies that should be carried out with precision in determining the role of vaccinations in post COVID-19 syndrome. In addition to this, these lingering symptoms were found to possibly vary with different variants of SARS-CoV-2. Yet determining the exact symptoms associated with specific variants and identifying the infectious variant in patients who have acquired these persistent symptoms remains a challenge [64]. Thus, more studies should focus on providing detailed explanations to clear the air regarding the link between long COVID symptoms and different variants of the virus.

## 5. Conclusions

Our review concluded that most of the COVID-19 patients who have recovered face COVID-19’s long-term effects and that the intensity of the prior COVID-19 affects each of these symptoms differently. After applying all exclusion criteria, a total of 33 papers, including cohort studies, cross-sectional studies and longitudinal studies were analyzed. All the articles suggested long-term consequences, ranging from immunological changes to basic symptoms such as fatigue and dyspnea.

Care and screening must be given to patients who have recovered from the infection. Guidelines for managing the long-term effects of long COVID-19 were jointly issued by the National Institute for Health and Care Excellence (NICE), the Scottish Intercollegiate Guidelines Network (SIGN) and the Royal College of General Practitioners (RCGP) [65], and these recommendations must be implemented by all healthcare facilities for better management. Setting up routine screening of COVID-19 recovered patients is necessary for the management of COVID-19’s long-term consequences. This ought to be a requirement for all healthcare facilities. A healthy lifestyle is also necessary to recover from the infection fully, in addition to screening.

## 6. Limitations

The main limitation of this review is that research on the long-term effects of COVID-19 is still nascent and has not been conducted on larger populations; thus, symptoms that persist beyond a year or two needs to be still identified. Our study has also not factored in the socio-demographic characteristics among reviewed articles as compared to peer scoping reviews, as it was not the objective of this review. Factoring these characteristics in the review could provide data on it being a determinant of persistence of long COVID-19. As the post COVID-19 symptoms are non-specific, a comparative analysis of signs and symptoms with the non-COVID-19 group, as studied in a systematic review by Mohamad Salim Alkodaymi et al., [66], will help with accurate diagnoses and treatment, which is lacking in our study. Furthermore, it is to be noted that the aftercare of patients with post COVID-19 syndrome has not been discussed in our study. This is because a very little amount of data is available on the internet regarding this topic, except for few studies that provide information about the rehabilitation and management [66,67]. Furthermore, as this study provides insight about various post COVID-19 symptoms in different populations, we did not elaborate specifically on every post COVID-19 symptom with regard to one or more systems, as analyzed in the systematic review by Thor Mertz Schou et al., [67].

## 7. Recommendations

Studies have been done that illustrate long-term consequences, but there are still many other factors that need to be taken into consideration. A key observation of our review is the lack of specific differences of symptoms reported according to the phase of long COVID-19 studied. This suggests that most symptoms persist beyond the initial phases. A key drawback assessed across studies were recall bias, selection bias and loss to follow-up; thus we recommend research with higher sample sizes, defined time points and multi-centric studies with good cohort systems be conducted. This can facilitate firm conclusions to be drawn for decision support symptoms to manage the long-term effects of post/chronic COVID-19 more effectively. We also recommend that the available scientific literature be matched to primary cohort data from hospital/research databases.

## Figures and Tables

**Figure 1:**
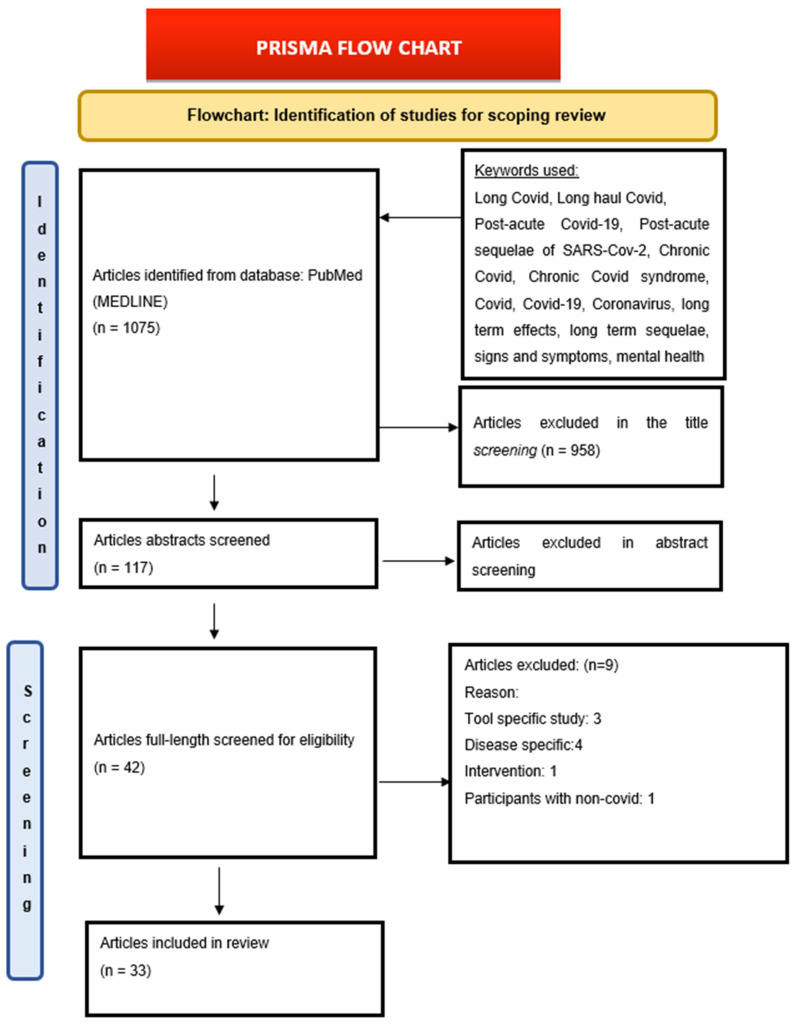
PRISMA Flow Chart.

**Table 2:** Result Analysis.

S.No	Major Step	Methods
3.1	Results of Search	The electronic search of the Pub Med (MEDLINE) database generated 1075 research articles using relevant keywords that can provide maximum information available based on inclusion and exclusion criteria. The authors of the current study then screened the articles by their titles and abstracts. They then analyzed the entire texts of selected research publications, and 33 research publications (Table 3) were selected and included in the review (Figure 1).
3.2	Listing of selected studies	The lists of studies discussed in this review are presented in Table 3 with the assigned study number that would be used as the study identifier (Study ID) throughout the study.

**Table 3:** Studies included in the review.

Study ID	Title	First Author
1	Multi organ impairment in low-risk individuals with post-COVID-19 syndrome: a prospective, community-based study [14].	Dennis, A.
2	Post COVID-19 squealae: A prospective observational study from Northern India [15].	Naik, S.
3	Post-acute COVID-19 Syndrome Negatively Impacts Physical Function, Cognitive Function, Health-Related Quality of Life, and Participation [16].	Tabacof, L.
4	Long-term sequelae are highly prevalent one year after hospitalization for severe COVID-19 [17].	Bellan, M.
5	Incidence and risk factors for persistent symptoms in adults previously hospitalized for COVID-19 [18].	Munblit, D.
6	Risk Factors Associated with Long COVID Syndrome: A Retrospective Study [19].	Asadi-Pooya, A.A.
7	Long-term clinical follow-up of patients suffering from moderate-to-severe COVID-19 infection: a mono centric prospective observational cohort study [20].	Darcis, G
8	Long-Term Evolution of Malnutrition and Loss of Muscle Strength after COVID-19: A Major and Neglected Component of Long COVID-19 [21].	Gérard, M.
9	Immunoglobulin signature predicts risk of post-acute COVID-19 syndrome [22].	Cervia, C.
10	Persistent symptoms and decreased health-related quality of life after symptomatic paediatric COVID-19: A prospective study in a Latin American tertiary hospital [23].	Fink, T.T.
11	Clinical characteristics with inflammation profiling of long COVID and association with 1-year recovery following hospitalization in the UK: a prospective observational study [24].	Evans, R.A.
12	Chronic Fatigue and Post exertional Malaise in People Living With Long COVID: An Observational Study [25].	Twomey, R.
13	Factors Associated with Post-Acute Sequalae of SARS-CoV-2 (PASC) After Diagnosis of Symptomatic COVID-19 in the Inpatient and Outpatient Setting in a Diverse Cohort [26].	Yoo, S.M.
14	Symptoms Experienced at the Acute Phase of SARS-CoV-2 Infection as Risk Factor of Long-term Post-COVID Symptoms: The LONG-COVID-EXP-CM Multicentre Study [27].	Fernández-de-Las-Peñas, C.
15	Predictors of chronic COVID-19 symptoms in a community-based cohort of adults [28].	Silverberg, J.I.
16	Post-acute COVID-19 condition in Saudi Arabia: A national representative study [29].	AlRadini, F.A.
17	Long COVID symptoms in SARS-CoV-2-positive children aged 0–14 years and matched controls in Denmark (LongCOVIDKidsDK): a national, cross-sectional study [30].	Kikkenborg Berg, S.
18	Post-sequalae one year after hospital discharge among older COVID-19 patients: A multi-centre prospective cohort study [31].	Fang, X.
19	The long-term functioning status of COVID-19 survivors: a prospective observational evaluation of a cohort of patients surviving hospitalization [32].	Battistella, L.R.
20	Long COVID-19 symptoms: Clinical characteristics and recovery rate among non-severe outpatients over a six-month follow-up [33].	Seang, S.
21	Health Status, Persistent Symptoms, and Effort Intolerance One Year After Acute COVID-19 Infection [34].	Kingery, J.R.
22	Associations between persistent symptoms after mild COVID-19 and long-term health status, quality of life, and psychological distress [35].	Han, J.H.
23	Long COVID 12 months after discharge: persistent symptoms in patients hospitalised due to COVID-19 and patients hospitalized due to other causes-a multicentre cohort study [36].	Rivera-Izquierdo, M.
24	Fatigue and Dyspnoea as Main Persistent Post-COVID-19 Symptoms in Previously Hospitalized Patients: Related Functional Limitations and Disability [37].	Fernández-de-Las-Peñas, C.
25	Risk factors for fatigue and impaired function eight months after hospital admission with COVID-19 [38].	Schouborg, L.B.
26	Prospective Evaluation of Autonomic Dysfunction in Post-Acute Sequela of COVID-19 [39].	Jamal, S.M.
27	Neurocognitive Profiles in Patients With Persisting Cognitive Symptoms Associated With COVID-19 [40].	Krishnan, K.
28	Long COVID in hospitalized and non-hospitalized patients in a large cohort in Northwest Spain, a prospective cohort study [41].	Pérez-González, A.
29	Sequelae, persistent symptomatology and outcomes after COVID-19 hospitalization: the ANCOHVID multicentre 6-month follow-up study [42].	Romero-Duarte, Á.
30	Six-Month Outcomes in Patients Hospitalized with Severe COVID-19 [43].	Horwitz, L.I.
31	Long-COVID’: a cross-sectional study of persisting symptoms, biomarker and imaging abnormalities following hospitalization for COVID-19 [44].	Mandal, S.
32	Physical, cognitive, and mental health impacts of COVID-19 after hospitalization (PHOSP-COVID): a UK multicentre, prospective cohort study [45].	Evans, R.A.
33	Post-acute sequelae of COVID-19: Evidence of mood and cognitive impairment [46].	Lamontagne, S.J.

**Table 4:** Study Characteristics.

Study ID	Title	First Author	Year of Study	Location	Study Design	Keywords	Study Population Age Group (in years)	Sample Size (No. of Patients)	Phase of COVID-19	Physiological Symptoms Assessed	Psychological Symptoms Assessed
1	Multi organ impairment in low-risk individuals with post-COVID-19 syndrome: a prospective, community-based study [14].	Dennis, A.	2021	UK	Observational study	COVID-19, epidemiology, health policy, public health	18–64	201	2	Fever, Headache, Cough, Dyspnea, Fatigue	-
2	Post COVID-19 sequelae: A prospective observational study from Northern India [15].	Naik, S.	2021	India	Observational study	COVID-19, long COVID-19, post COVID-19 sequelae, post COVID-19 syndrome	18–64	1234	2	Chest Pain, Myalgia Insomnia, Cough, Dyspnea, Fatigue	Depression, Mood Disorders, Brain Fog
3	Post-acute COVID-19 Syndrome Negatively Impacts Physical Function, Cognitive Function, Health-Related Quality of Life, and Participation [16].	Tabacof, L.	2022	USA	Observational study	Post-acute COVID-19, fatigue, cognition, employment, quality of life	18–64	156	3	Loss Of Appetite, Sweating, Tinnitus, Sexual Dysfunction, Neuropathic Pain, Abdominal Pain, Sore Throat, Dizziness, Vertigo, Syncope, Visual Impairment, Nausea, Hair loss, Diarrhea, Palpitation, Arthralgia, Chest Pain, Insomnia, Headache, Dyspnea, Fatigue	Memory Loss, Forgetfulness, Confusion
4	Long-term sequelae are highly prevalent one year after hospitalization for severe COVID-19 [17].	Bellan, M.	2021	Italy	Observational study	microbiology virology	18–64	238	3	Hair Loss, Arthralgia, Anosmia, Chest Pain, Myalgia, Cough, Dyspnea, Fatigue	-
5	Incidence and risk factors for persistent symptoms in adults previously hospitalized for COVID-19 [18].	Munblit, D.	2021	Russia	Clinical Trial	COVID-19, PAASC, asthma, long COVID-19, post COVID-19 Condition, Post COVID-19 syndrome, Post acute sequelae, SARS-CoV-2 infection, risk factor	18–64	2649	3	Vision Impairment, Hair loss, Myalgia Insomnia, Dyspnea, Fatigue	Memory Loss, Forgetfulness
6	Risk Factors Associated with Long COVID-19 Syndrome: A Retrospective Study [19].	Asadi-Pooya, A.A.	2021	Iran	Multicentric study	COVID-19, medicine, SARA-CoV-2 Viruses	18–64	4681	2	Exertional Intolerance, Myalgia, Dyspnea, Fatigue	-
7	Long-term clinical follow-up of patients suffering from moderate-to-severe COVID-19 infection: a monocentric prospective observational cohort study [20].	Darcis, G.	2021	Belgium	Observational study	COVID-19, long COVID-19, post COVID-19, post- acute COVID-19, sequelae	18–64	199	2	Hair Loss, Chest Pain, Cough, Dyspnea, Fatigue	-
8	Long-Term Evolution of Malnutrition and Loss of Muscle Strength after COVID-19: A Major and Neglected Component of Long COVID-19 [21].	Gérard, M.	2021	England	Cohort study	Cohort study, intensive care unit, long COVID-19, malnutrition, muscle strength, obesity, performance status, self-evaluation.	18–64	549	3	Malnutrition, Palpitation	Memory Loss, Forgetfulness, Depression, Mood Disorders, Anxiety, Concentration Cognitive Impairment, Post Traumatic Stress,
9	Immunoglobulin signature predicts risk of post-acute COVID-19 syndrome [22].	Cervia, C.	2022	Switzerland	Observational study	Not mentioned	18–64	175	3	Anosmia, Dyspnea, Fatigue, Ageusia	Depression, Mood Disorders, Anxiety
10	Persistent symptoms and decreased health-related quality of life after symptomatic pediatric COVID-19: A prospective study in a Latin American tertiary hospital [23].	Fink, T.T.	2021	Brazil	Observational study	Long corona virus disease 2019, child, adolescent, sequelae, multisystem inflammatory syndrome in children	<18	105	2	Anemia, Arthralgia, Myalgia, Insomnia, Headache, Dyspnea	Concentration and Cognitive Impairment
11	Clinical characteristics with inflammation profiling of long COVID-19 and association with 1-year recovery following hospitalization in the UK: a prospective observational study [24].	Evans, R.A.	2022	UK	Observational study	Not mentioned	18–64	2320	Mixed	Exertional Intolerance, Arthralgia, Insomnia, Dyspnea, Fatigue, Ageusia	Memory Loss, Forgetfulness, Slowed Down Thinking
12	Chronic Fatigue and Post exertional Malaise in People Living With Long COVID-19: An Observational Study [25].	Twomey, R.	2022	USA	Observational study	COVID-19, exercise tolerance, fatigue, long COVID-19, patient-reported outcomes, rehabilitation.	18–64	213	1	Loss Of Appetite, Sore Throat, Rashes, Dizziness, Vertigo, Vision Impairment, Nausea, Diarrhea, Fever Palpitation, Ageusia, Myalgia, Headache, Cough, Dyspnea, Fatigue	Brain Fog
13	Factors Associated with Post-Acute Sequelae of SARS-CoV-2 (PASC) After Diagnosis of Symptomatic COVID-19 in the Inpatient and Outpatient Setting in a Diverse Cohort [26].	Yoo, S.M.	2022	USA	Observational study	Not mentioned	18–64	1038	1	Sinus, Rashes, Fever, Anosmia, Chest Pain, Ageusia, Dyspnea, Fatigue	Memory Loss, Forgetfulness, Brain Fog
14	Symptoms Experienced at the Acute Phase of SARS-CoV-2 Infection as Risk Factor of Long-term Post-COVID-19 Symptoms: The LONG-COVID-19-EXP-CM Multicentre Study [27].	Fernández-de-Las-Peñas, C.	2022	Spain	Multicentric study	COVID-19, acute phase, persistent, risk factors symptoms	18–64	1969	3	Sore Throat, Rashes, Vision Impairment, Diarrhea, Palpitation, Anosmia, Ageusia, Dyspnea, Fatigue	-
15	Predictors of chronic COVID-19 symptoms in a community-based cohort of adults [28].	Silverberg, J.I.	2022	USA	Observational study	Not mentioned	18–64	390	Mixed	Abdominal Pain, Headache, Fatigue	-
16	Post-acute COVID-19 condition in Saudi Arabia: A national representative study [29].	AlRadini, F.A.	2022	Saudi Arabia	Multicentric study	Late symptoms, resistant symptoms, post-acute COVID-19, SARS-CoV-2	18–64	225	1	Fever, Arthralgia, Anosmia, Ageusia, Headache, Dyspnea, Fatigue	-
17	Long COVID-19 symptoms in SARS-CoV-2-positive children aged 0–14 years and matched controls in Denmark (LongCOVID19 KidsDK): a national, cross-sectional study [30].	Kikkenborg Berg, S.	2022	Denmark	Observational study	Not mentioned	<18	10997	1	Arthralgia, Anosmia, Chest Pain, Myalgia Cough, Dyspnea, Fatigue	-
18	Post-sequelae one year after hospital discharge among older COVID-19 patients: A multi-centre prospective cohort study [31].	Fang, X.	2022	China	Multicentric study	COVID-19, sequelae, older people, Wuhan, SARS-CoV-2	>64	1233	3	Sweating, Palpitations, Anosmia, Chest Pain, Ageusia, Myalgia, Cough Fatigue	Anxiety
19	The long-term functioning status of COVID-19 survivors: a prospective observational evaluation of a cohort of patients surviving hospitalization [32].	Battistella, L.R.	2022	Italy	Multicentric study	COVID-19, rehabilitation, medicine, respiratory infections	18–64	149	3	Excessive Sleep, Exertional Intolerance, Insomnia, Dyspnea	Depression and More Disorders
20	Long COVID-19 symptoms: Clinical characteristics and recovery rate among non-severe outpatients over a six-month follow-up [33].	Seang, S.	2022	France	Observational study	Long COVID-19, outpatients, post infectious disorders, recovery	18–64	36	3	Fever, Arthralgia, Anosmia, Chest Pain, Ageusia, Myalgia, Headache, Cough, Dyspnea	-
21	Health Status, Persistent Symptoms, and Effort Intolerance One Year After Acute COVID-19 Infection [34].	Kingery, J.R.	2022	USA	Observational study	COVID-19, PASCA, Persistent symptoms	18–64	530	3	Numbness, Abdominal Pain, Diarrhea, Headache, Dyspnea, Fatigue	Brain Fog
22	Associations between persistent symptoms after mild COVID-19 and long-term health status, quality of life, and psychological distress [35].	Han, J.H.	2022	USA	Multicentric study	COVID-19, outcomes, long COVID-19 postacute sequalae of COVID-19, PASC, post COVID-19conditions	18–64	2092	3	Hair loss, Exertional Intolerance, Chest Pain, Ageusia, Insomnia, Fatigue, Dyspnea	Concentration and Cognition Impairment
23	Long COVID-19 12 months after discharge: persistent symptoms in patients hospitalized due to COVID-19 and patients hospitalized due to other causes-a multicentre cohort study [36].	Rivera-Izquierdo, M.	2022	Spain	Multicentric study	Cohort, follow up, long term, persistent COVID-19, sequalae	18–64	163	3	Arthralgia, Insomnia, Headache, Fatigue	Memory Loss, Forgetfulness, Depression, Mood Disorders, Anxiety, Confusion
24	Fatigue and Dyspnea as Main Persistent Post-COVID-19 Symptoms in Previously Hospitalized Patients: Related Functional Limitations and Disability [37].	Fernández-de-Las-Peñas, C.	2022	Spain	Multicentric study	COVID-19, dyspnea, fatigue, function, risk factors	18–64	1142	3	Diarrhoea, Palpitation, Chest Pain, Cough, Fatigue, Dyspnea	-
25	Risk factors for fatigue and impaired function eight months after hospital admission with COVID-19 [38].	Schouborg, L.B.	2022	Denmark	Observational study	Not mentioned	18–64	83	3	Loss Of Functional Status Fatigue	-
26	Prospective Evaluation of Autonomic Dysfunction in Post-Acute Sequela of COVID-19 [39].	Jamal, S.M.	2022	USA	Observational study	HUTT, neurocardiogenic syncope, orthostatic hypotension, intolerance, POTS	18–64	24	2	Palpitations, Exertional Intolerance, Chest Pain, Headache, Fatigue	Concentration and Cognition Impairment
27	Neurocognitive Profiles in Patients With Persisting Cognitive Symptoms Associated With COVID-19 [40].	Krishnan, K.	2022	USA	Observational study	Brain fog, COVID-19, cognition, long COVID-19, neuropsychology	18–64	20	2	Dizziness, Vertigo, Nausea, Diarrhea, Palpitation, Exertional Intolerance, Arthralgia, Insomnia, Cough, Dyspnea, Fatigue	Memory Loss, Forgetfulness, Brain Fog, Concentration and Cognition Impairment
28	Long COVID-19 in hospitalized and non-hospitalized patients in a large cohort in Northwest Spain, a prospective cohort study [41].	Pérez-González, A.	2022	Spain	Clinical trial	Not mentioned	18–64	248	3	Hair Loss, Anosmia, Ageusia, Myalgia, Insomnia, Headache, Cough, Dyspnea, Fatigue	-
29	Sequelae, persistent symptomatology and outcomes after COVID-19 hospitalization: the ANCOHVID multicentre 6-month follow-up study [42].	Romero-Duarte, Á.	2021	Spain	Observational study	COVID-19, long COVID-19, post discharge, sequelae, persistent symptoms, primary care, follow up	18–64	797	3	Fever, Numbness, Malnutrition, Anemia, Constipation, Rib Pain, Dysuria, Hemautria, Oliguria, Exertional Intolerance Abdominal Pain, Nausea, Glycemia, Renal Problems, Rashes, Hair loss, Diarrhea, Dizziness, Vertigo Or Syncope, Vision Impairment, Anosmia, Myalgia, Insomnia, Cough, Fatigue	Anxiety and Confusion
30	Six-Month Outcomes in Patients Hospitalized with Severe COVID-19 [43].	Horwitz, L.I.	2021	USA	Observational study	COVID-19, long COVID-19, patient reported outcomes, post discharge outcomes	18–64	152	3	Numbness, Tinnitus, Rashes, Dizziness, Vertigo Or Syncope, Vision Impairment, Nausea, Palpitations, Anosmia, Insomnia, Headache, Dyspnea, Fatigue	Memory Loss, Forgetfulness, Brain Fog, Concentration and Cognition Impairment
31	Long-COVID-19: a cross-sectional study of persisting symptoms, biomarker and imaging abnormalities following hospitalization for COVID-19 [44].	Mandal, S.	2021	UK	Observational study	Respiratory infection, viral infection	18–64	348	1	Dyspnea, Fatigue, Cough	Depression and Mood Disorders
32	Physical, cognitive, and mental health impacts of COVID-19 after hospitalization (PHOSP-COVID): a UK multicentre, prospective cohort study [45].	Evans, R.A.	2021	UK	Multicentric study	Not mentioned	18–64	1077	2	Exertional Intolerance, Arthralgia, Insomnia, Dyspnea, Fatigue	Memory Loss, Forgetfulness, Slowed Down Thinking
33	Post-acute sequelae of COVID-19: Evidence of mood & cognitive impairment [46].	Lamontagne, S.J.	2021	Canada	Multicentric study	Corona virus disease, SARS-CoV-2, PASC, ANT, inflammation, cognitive control, anhedonia, depression,	18–64	50	1	Sinus, Sore Throat Nausea, Diarrhea, Fever, Ageusia, Headache, Cough, Dyspnea, Fatigue	-

**Table 5:** Study Design.

Study Design	Number of Studies(%)	Study ID
Observational study	20 (60.6)	1, 2, 3, 4, 6, 7, 10, 11, 12, 13, 15, 17, 20, 21, 25, 26, 27, 29,30, 31
Multicentric study	10 (30.3)	9, 14, 16, 18, 19, 22, 23, 24, 32, 33
Clinical Trials	2 (6)	5, 28
Cohort Study	1 (3)	8

**Table 6:** Study location Income classification.

Study location Income Classification	Number of Studies = *n* (%)	Study ID
High-income economies	27 (81.8)	1, 3, 4, 7, 8, 9, 11, 12, 13, 14, 15, 16, 17, 20, 21, 22, 23, 24, 25, 26, 27, 28, 29, 30, 31, 32, 33
Upper-middle income	4 (12.12)	5, 10, 18, 19
Lower-middle income	2 (6)	2, 6

**Table 7:** Distribution of Study Population among studies.

Study Population	Number of Studies = *n* (%)	Study ID
Children	2 (6%)	10, 17
Elderly	1 (3%)	18
Adult	30 (91%)	All other studies

**Table 8:** Distribution of Physiological symptoms identified.

Physiological Symptoms	Total Studies = (*n*) (%)	Study IDs
Performance and Perceived Fatigue/tiredness/weakness	29 (87)	1, 2, 3, 4, 5, 6, 7, 9, 11, 12, 13, 14, 15, 16, 17, 18, 21, 22, 23, 24, 25, 26, 27,28, 29, 30, 31, 32, 33
Shortness of breath/Dyspnea/Breathlessness	26 (78)	1, 2, 3, 4, 5, 6,7, 9, 10, 11, 12, 13, 14,16,17, 19, 20, 21, 22, 24, 27, 28, 30, 31, 32, 33
Cough	15 (45)	1, 2, 4, 7, 12, 15, 17, 18, 20, 24, 27, 28, 29, 31, 33
Headache	13 (39)	1, 3, 10, 12, 15, 16, 20, 21, 23, 26, 28, 30, 33
Insomnia	13 (39)	2, 3, 5, 10, 11, 19, 22, 23, 27, 28, 29, 30, 32
Myalgia	12 (36)	2, 4, 5,6, 10, 17, 18, 20, 28, 29, 33, 12
Ageusia/loss of taste	12(36)	9, 11, 12, 13, 14, 16, 18, 20, 22, 28, 30, 33
Chest pain/tightness	11 (33)	2, 3, 4, 7, 13, 17, 18, 20, 22, 24, 26
Anosmia/loss of smell	11(33)	4, 9, 13, 14, 16, 17, 18, 20, 28, 29,30
Arthralgia	10 (30)	3, 4, 10, 11, 16, 17, 20, 23, 27, 32
Exertional intolerance	8 (24)	6, 11, 26. 27, 19, 22, 29, 32
Palpitation	8 (24)	3, 8, 12, 14, 18, 24, 26, 27, 30
Fever	7 (21)	1, 12, 13, 16, 20, 29, 33
Diarrhoea	8 (24)	3, 12, 14, 21, 24, 27, 29, 33
Hair loss	7 (21)	3, 4, 5, 7, 22, 28, 29
Nausea/vomiting	6 (18)	3, 12, 27, 29, 30, 33
Vision impairment/eye problems	6 (18)	3, 5, 12, 14, 29, 30
Dizziness/vertigo/syncope	5 (15)	3, 12, 27, 29, 30
Rashes in skin/skin disorders	5 (15)	12, 13, 14, 29, 30
Sore throat/throat pain	4 (12)	3, 12, 14, 33
Abdomen pain	4 (12)	3, 15, 21, 29
Numbness/paresthesia	3 (9)	21, 29, 30
loss of appetite	2 (6)	3, 12
Malnutrition	2 (6)	8, 29
Anemia	2 (6)	10, 29
Sweating	2 (6)	3, 18
Tinnitus	2 (6)	3, 30
Sinus/nasal congestion	2 (6)	12, 33
Sexual dysfunction	1(3)	3
Excessive sleepiness	1 (3)	19
Neuropathic pain	1 (3)	3
Constipation	1 (3)	39
Loss of functional status	1 (3)	25
Rib pain/thoracic pain	1 (3)	29
Dysuria, haematuria, oliguria	1 (3)	29
Glycaemia/renal problems	1 (3)	29

**Table 9:** Distribution of Psychological symptoms identified.

Psychological Symptoms	Total Studies, *n* (%)	Study IDs
Memory loss/forgetfulness	9 (27)	3, 5, 8, 11, 14, 23, 27, 30, 32
Depression and Mood disorders	7 (21)	2, 3, 8, 9, 19, 23, 31
Brain fog	6 (18)	2, 12, 14, 21, 27, 30
Anxiety	6 (18)	8, 9, 18, 19, 23, 29
Concentration/cognitive impairment	6 (18)	8, 10, 22, 26, 27, 30
Confusion	3 (9)	3, 23,29
Slowdowned thinking	2 (6)	11, 32
Post-traumatic stress	1 (3)	8

**Table 10:** A summary of major limitations.

Phase	No. of Studies	Study ID	Major Limitations
1	6	12, 13, 16, 17, 31, 33	Selection bias: People presenting with symptoms were more likely to participate. Mothers of children with a greater number of symptoms are more inclined to join. No comparative group of participants representing non-hospitalised patients. Recall bias. Based on the purely behavioural nature of the study, precise mechanisms underlying the link between COVID-19 and depression remain unknown.
2	8	1, 2, 6, 7, 10, 26, 27, 32	Incidental findings were possible in asymptomatic individuals. The study population was not ethnically diverse. Selection bias: the study was only from tertiary hospitals; four studies reported this limitation. Phone data collection. Loss to follow up. Median of 4 months assessed; need long-term follow up. Relatively small sample size.
3	17	3, 4, 5, 8, 9, 14, 18, 19, 20, 21, 22, 23, 25, 24, 28, 29, 30	The lack of validated scales to measure most of the symptoms. Relatively small sample size. Failed to show a clear association between severity of symptoms. Retrospective studies have recall bias. Most studies used subjective methods to assess symptoms and not objective measures; for example, pulmonary tests, blood oxygen saturation, inflammatory biomarkers or chest X-rays. Telephonic data collection; the absence of a control group for comparison. Loss to follow up.
Mixed	2	11, 15	Selection bias for participants returning for a 1-yearvisit.

## Data Availability

The data that supports this study are available upon request from the corresponding author.
